# The complete mitochondrial genomes of three geographical lineages in short ninespine stickleback (*Pungitius kaibarae*) complex and their phylogenetic implication

**DOI:** 10.1080/23802359.2017.1361362

**Published:** 2017-08-02

**Authors:** Han-Gyu Bae, Yoon Jeong Lee, Hyung-Bae Jeon, Dong-Young Kim, Hari Won, Seulki Park, Junghwa An, Ho Young Suk

**Affiliations:** a Department of Life Sciences, Yeungnam University, Gyeongsan-si, South Korea;; b National Institute of Biological Resources, Environmental Research Complex, Incheon, South Korea

**Keywords:** *Pungitius kaibarae*, short ninespine stickleback, divergence time, phylogenetics, gasterosteid

## Abstract

The short ninespine stickleback, *Pungitius kaibarae*, is a small gasterosteid species complex containing three geographical lineages: ND, NE and SE. Here, complete mitochondrial genomes of these three lineages were analyzed to estimate the genetic differentiation among them and to identify their phylogenetic placement in genus *Pungitius*. Although the overall genome structure was identical among those three lineages, the genome sizes were slightly different from each other, ranging from 16,489 to 16,500 bp. Upon robust phylogenetic tree inferred by Bayesian algorithm, ND and SE showed relatively higher affinity, and those three lineages formed a monophyletic group with Russian *P. tymensis*, clearly supporting previous studies

Short ninespine stickleback, *Pungitius kaibarae*, is a small gasterosteid fish, native to the east coast on the Korean Peninsula, the southeast tip of Russia (Bae and Suk 2015) and northern west coast in Honshu (Japan; Takahashi et al. 2016). On the Korean Peninsula, this species is observed in three geographically separated regions: NE, SE and ND (Bae and Suk 2015). In genetic analyses using mitochondrial and microsatellite loci, a substantial differentiation was detected among those three regional populations (Bae and Suk 2015). We characterized the complete mitochondrial genomes of those three geographical lineages and performed phylogenetic examination using their mitochondrial genomes to estimate the genetic differentiation among them and to identify their phylogenetic placement in genus *Pungitius*. Genomic DNA was extracted from the fin clips collected from three different localities (NE: Yeongok River, E37°84′70.87′′, N128°81′06.73′′; SE: Hyeongsan River, E35°90′29.04′′, N129°25′86.75′′; ND: Nakdong River, E36°01′81.80′′, N128°97′93.17′′; Bae and Suk 2015). The fish and genomic DNA samples were stored in collection storage at the Department of Life Sciences, Yeungnam University as the voucher numbers, YUSS552 – −554 and YUSS-PK01 – −03, respectively. The mitochondrial genomes were amplified using the primers designed based on the congeneric complete genomes stored in NCBI and were commercially sequenced on an ABI 3730XL DNA Analyzer (Applied Biosystems, Foster City, CA). The fragment sequences were assembled to consensus mitochondrial genomes using Geneious v6.05 (Kearse et al. 2012; http://www.geneious.com).

The loci were annotated using MITOS web server (Bernt et al. 2013) and the complete mitogenome sequences were deposited at NCBI GenBank under the accession numbers KY628301, KY628302 and KY628303. The whole sequences of NE, SE and ND were 16,500, 164,090 and 16,489 bp, respectively, and included 13 protein-coding genes, 2 ribosomal RNA genes, 22 transfer RNA genes and 2 non-coding regions (O_L_ and D-loop). Every protein-coding gene contained ATG start codon with a single exception in COX1 starting with GTG. Incomplete stop codon with single nucleotide T was detected at three coding genes, COX2, Cytb and ND4. The origin of L-strand (O_L_) was detected in the cluster of five tRNA genes, with being located between tRNA^Asn^ and tRNA^Cys^. The gene arrangement of three mitogenomes was identical to those observed in other *Pungitius* species (Kawahara et al. 2009; Hwang et al. 2012a, 2012b; Guo et al. 2016a, 2016b; Shikano et al. 2016a, 2016b).

The evolutionary relationship among *P. kaibarae* lineages and the phylogenetic placement of this complex within genus *Pungitius* were inferred based on 13 protein-coding and 2 rRNA genes using BEAST2 (Bouckaert et al. 2014) implemented following the parameter setting and time calibration described in Wang et al. (2015). The overall topology was nearly identical to those constructed in previous studies (Bae and Suk 2015; Wang et al. 2015; Takahashi et al. 2016), despite slight differences in the timing of divergence among major clades. In the tree, ND individuals showed relatively higher phylogenetic affinity to SE than NE (Figure 1). *P. kaibarae* complex formed a monophyletic group with Russian *P. tymensis*, indicating that three lineages originated from the southward migration (Bae and Suk 2015). Our study may be useful to reconstruct the consensus phylogenetic tree that is essential for the future study of biogeographic dispersal and geographic isolation of these lineages.

**Figure 1.**
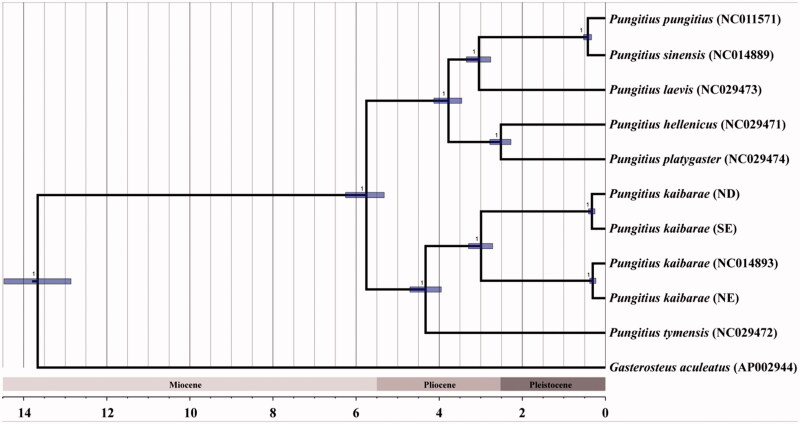
Bayesian inference tree reconstructed using mitochondrial genomes of genus *Pungitius*, based on the parameter setting and time calibration described in Wang et al. (2015). NCBI GenBank accession numbers were indicated for the data retrieved.
